# Frequency of Renal Function Parameter Abnormalities in Patients with Psoriatic Arthritis and Rheumatoid Arthritis: Real-World Evidence from Clinical Practice

**DOI:** 10.3390/jcm11041029

**Published:** 2022-02-16

**Authors:** Fabiola Atzeni, Pietro Muto, Javier Rodríguez-Carrio, Ignazio Francesco Masala

**Affiliations:** 1 Rheumatology Unit, Department of Experimental and Internal Medicine, University of Messina, 98168 Messina, Italy; pietro.muto87@gmail.com; 2 Department of Functional Biology, Immunology Area, Faculty of Medicine, University of Oviedo, 33006 Oviedo, Spain; rodriguezcjavier@uniovi.es; 3 Instituto de Investigación Sanitaria del Principado de Asturias (ISPA), 33011 Oviedo, Spain; 4 Trauma and Orthopedic Unit, Santissima Trinità Hospital, 09121 Cagliari, Italy

**Keywords:** psoriatic arthritis, rheumatoid arthritis, kidney, renal dysfunction, remission

## Abstract

Objective: Patients with psoriatic arthritis (PsA) or rheumatoid arthritis (RA) commonly develop renal dysfunction due to either systemic inflammation or drug-related nephrotoxicity. This study compared renal function parameters in patients with PsA versus those with RA and examined the impact of clinical remission or disease relapse on renal function. Methods: This single-center retrospective study was conducted at the University Hospital of Messina, Italy. Adult patients (aged ≥18 years) with PsA or RA who attended the rheumatology clinic within the past 6 months were identified from electronic medical records. Results: In total, 45 patients with PsA (*n* = 23) or RA (*n* = 22) were included. The mean (standard deviation) age was 55.6 (15.9) years, and 78% of participants were female. Patient age, renal function, and medical history were generally similar between the two disease groups, although significantly more RA patients were smokers, and more PsA patients had comorbid hypertension. The prevalence of estimated glomerular filtration rate [eGFR] ≤90 mL/min/1.73 m^2^ at 1, 6, and 12 months of treatment ranged from 38.5% to 58.3% in the PsA group and from 45.5% to 54.5% in the RA group and did not significantly differ between disease groups. Clinical remission did not appear to affect renal function parameters in either disease group; however, relapse was associated with significantly higher serum creatinine levels in PsA patients at the same timepoint. Conclusion: In this study, patients with PsA and RA had a similar prevalence of renal function parameter abnormalities over 12 months of treatment. Disease relapse may impact renal function in patients with PsA.

## 1. Introduction

Patients with rheumatoid arthritis (RA) commonly develop renal involvement, which may be disease-related (i.e., associated with systemic inflammation) or drug-related (i.e., occurring as a result of nephrotoxicity) [1,2]. Drug-induced nephrotoxicity commonly develops with some older conventional disease-modifying antirheumatic drugs (cDMARDs), such as penicillamine, cyclosporine, and gold, as well as with nonsteroidal anti-inflammatory drugs (NSAIDs), although it has become less prevalent with the availability of less nephrotoxic cDMARDs and biologic agents [1]. Several regions of the kidney, including the glomerulus, interstitium, and vasculature, can be affected by RA (i.e., the disease per se) and/or by RA medications; however, the majority of RA patients with nephropathy have mesangial, membranous, or crescentic glomerulonephritis [1].

A common cause of renal involvement in patients with RA is amyloidosis [3]. In fact, in several reports of patients with amyloidosis, RA was the second most common cause of amyloidosis after infections [4,5]. However, the incidence of amyloidosis has significantly decreased over the years [6] because RA is treated earlier and more aggressively. It has been suggested that antitumor necrosis factor (TNF) agents in particular may prevent or ameliorate amyloidosis [7,8].

Renal disease can also occur in patients with psoriatic arthritis (PsA) but at a lower prevalence than in RA patients [9]. In patients with autoimmune inflammatory disease, the development of renal dysfunction is a concern for a number of reasons, including the link between chronic kidney disease (CKD) and cardiovascular disease (CVD) [10,11,12], as well as the potential impact of renal impairment on the pharmacokinetics of some medications [13]. In cross-sectional studies conducted in the United Kingdom and Japan, traditional CV risk factors, such as hyperlipidemia, hypertension, age, and insulin resistance, were associated with CKD development, whereas RA disease activity, severity, and duration were not [14,15].

To date, few studies have compared the clinical characteristics of patients with RA and PsA who develop renal dysfunction during treatment [16], and there are limited data on the relationship between renal dysfunction and cardiovascular risk factors in these two groups. The aim of the current study was (i) to compare renal function parameters in patients with RA versus those with PsA and (ii) to evaluate the impact of disease remission and relapse on renal function parameters.

## 2. Patients and Methods

### 2.1. Study Design and Patient Population

This single-center retrospective observational study was conducted with PsA and RA patients attending the Rheumatology Unit of the University Hospital of Messina, Italy. Researchers at the University Hospital reviewed electronic medical records and identified patients who met the following inclusion criteria: adult patients (aged ≥ 18 years) who attended the clinic for routine examinations during the last 6 months; had a definite diagnosis of PsA or RA, according to the CASPAR criteria for PsA [17] and the American College of Rheumatology/European League Against Rheumatism 2010 criteria for RA [18]; and received appropriate medication after the first visit and had renal function evaluations every 3–6 months. Exclusion criteria were a body mass index (BMI) > 35 kg/m^2^; a history of previous CKD or recent renal disease; a previous diagnosis of crystal deposit arthropathy (gout); or a history of cancer, lymphoproliferative disease, uncontrolled diabetes, unstable ischemic heart disease, or congestive heart failure.

Data collected were patient history, self-assessment questionnaires, and the findings of physical examinations and laboratory investigations. Patient history included age; sex; BMI; disease duration; family and personal history of psoriasis; comorbidities; and use of NSAIDs, corticosteroids, and conventional and/or biologic DMARDs.

All patients gave informed consent to participate in the study, which was conducted in accordance with the Declaration of Helsinki and local regulations. Local ethics committee approval was not required, as the participants underwent clinical and clinimetric examinations according to routine protocols used at the recruiting hospital.

### 2.2. Clinical Assessments

Clinical assessments included the number of tender and swollen joints, the duration of morning stiffness, erythrocyte sedimentation rate (ESR), and C-reactive protein (CRP) levels. Radiographs of the hands and wrists were obtained for all participants. Disease activity was assessed using the criteria of disease activity score in 28 joints (DAS28) [19]. According to the DAS28, disease activity was interpreted as remission (DAS28 < 2.6), low (DAS28 ≥ 2.6 to ≤3.2), moderate (DAS28 > 3.2 to ≤5.1), or high (DAS28 > 5.1). Relapse was defined as any worsening of DAS28 score during follow-up.

### 2.3. Biochemical Assessments

The laboratory variables relevant to RA activity (i.e., ESR, white blood cell and platelet counts, and CRP levels) were measured using standard laboratory assays. Immunoglobulin M rheumatoid factor (RF) was measured by immunonephelometry using a quantitative N latex RF system (Dade Behring, Marburg, Germany), with RF titers >15 IU/mL considered positive. Anticyclic citrullinated peptide autoantibodies were tested using a commercially available second-generation enzyme-linked immunosorbent assay kit (Menarini Diagnostics, Florence, Italy).

Estimated glomerular filtration rates (eGFR) were calculated using the Cockroft–Gault formula [20]. Other standard clinical laboratory tests were performed under fasting conditions on the same day, as per standard procedures.

Creatinine levels and eGFR at 1, 3, 6, and 12 months were extracted for this analysis, as well as data at the last available assessment if this did not correspond to one of those timepoints.

### 2.4. Statistical Analysis

Patient characteristics were summarized using frequencies and percentages for dichotomous variables and mean (standard deviation [SD]) for continuous variables. The frequency of dichotomous variables in each disease group (PsA and RA) was compared using a two-sided Fisher’s exact test or Pearson chi-squared test, and the distribution of continuous variables between groups was compared using the Mann–Whitney U test. The Mann–Whitney U test was also used to compare the distribution of continuous renal function variables within each disease group, between patients with and without disease remission, and between those with and without relapse. In particular, we sought associations between patients’ renal function parameters and remission/relapse status assessed at the same timepoint. A *p*-value < 0.05 was considered significant. The null hypothesis was not rejected when the distribution of variables between groups (with versus without remission, with versus without relapse) was not significantly different (*p* > 0.05), based on an asymptotic analysis. Linear regression analysis was used to assess whether sex was a confounder in the context of differences in mean height, weight, and BMI between PsA and RA patients. To investigate the impact of potential confounders on creatinine levels (dependent variable), we also conducted a multivariate linear regression analysis with sex, BMI, and hypertension as covariates (independent variable).

## 3. Results

### 3.1. Study Participants

Forty-five patients with PsA (*n* = 23) or RA (*n* = 22) met the criteria for inclusion in the study. The majority of participants were female (*n* = 35; 78%); 17/23 in the PsA group and 18/22 in the RA group were women. The mean (SD) age was 55.6 (15.9) years in the overall cohort and was not significantly different between patients with PsA and RA (Table 1). The distribution of weight, height, and BMI was significantly different between patients in the PsA group and those in the RA group (Table 2). A sex-adjusted linear regression model confirmed that patients with PsA were significantly taller (*p* = 0.015), weighed more (*p* = 0.001), and thus had higher BMI (*p* = 0.01) than patients with RA. The finding of higher weight, BMI, and prevalence of hypertension in PsA patients compared with RA patients is consistent with the high incidence of CV risk factors among patients with PsA reported in literature [21].

Participants in the two disease groups were comparable regarding past medical history, but significantly more patients in the RA group were smokers, whereas significantly more patients in the PsA group had comorbid hypertension (Table 1).

The majority of the patients (75%) in both disease groups were treated with methotrexate combined with anti-TNF drugs. Other biologic therapies in the PsA group included anti-interleukin (IL)-17 therapy in five patients, and in the RA group, abatacept in four patients, anti-IL-6 therapy in two patients, and rituximab in two patients. Thirteen patients in the RA group and one in the PsA were treated with corticosteroids (mean [SD] daily dose, 2.5 [5.0] mg). All patients were treated with on-demand NSAIDs, but none was taking NSAIDs daily.

### 3.2. Renal Function

**Figure 1:**
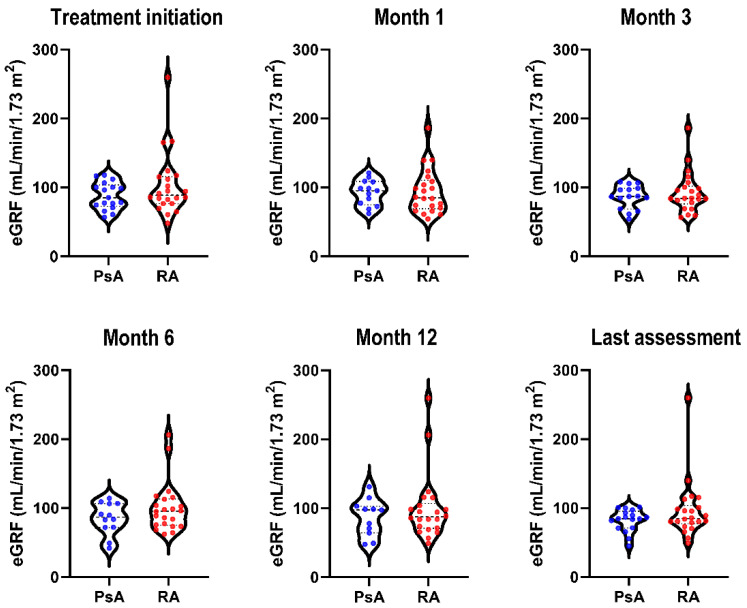
Distribution of estimated glomerular filtration rate in patients with psoriatic arthritis (PsA) or rheumatoid arthritis (RA) over 12 months of treatment. eGFR, estimated glomerular filtration rate.

**Figure 2:**
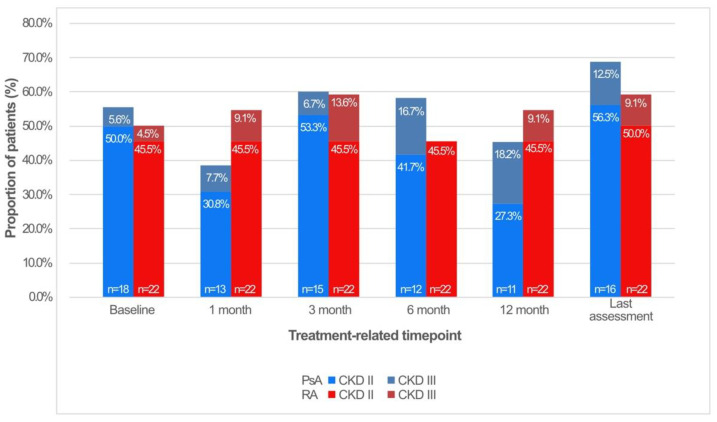
Percentage of patients with psoriatic arthritis (PsA) or rheumatoid arthritis (RA) who had an abnormal estimated glomerular filtration rate ≤90 mL/min/1.73 m^2^ at each timepoint over 12 months of treatment. Based on their eGRF levels, patients are stratified according to chronic kidney disease stages (CKD). The percentage was calculated as the proportion of patients with abnormal eGFR within a given disease group at a given timepoint.

There was no significant difference in mean serum creatinine levels or estimated GFR (eGFR) between patients in the PsA and RA groups at any timepoint (Table 3 and Figure 1). The proportion of patients with renal dysfunction (i.e., eGFR ≤ 90 mL/min/1.73 m^2^) ranged from 38.5% to 58.3% in the PsA group and from 45.5% to 54.5% in the RA group from treatment initiation to up to 12 months of treatment. At all measured timepoints, the prevalence of renal function parameter abnormalities was not significantly different between the two disease groups (Figure 2). Based on eGFR values, we stratified patients into chronic kidney disease (CKD) stages (Figure 2 and Supplementary Figure S1). There were no patients included in stage IV, and only a minor proportion was classified as stage III. There was no association between creatinine levels and gender, BMI, or arterial hypertension (Supplementary Tables S1 and S2).

### 3.3. Impact of Disease Remission or Relapse on Renal Function Parameters

Clinical remission was achieved during treatment in 16 patients (69.6%) in the PsA group and 10 (45.5%) in the RA group.

In the PsA group, the distribution of mean eGFR and serum creatinine levels was not significantly different between patients with versus without remission at the same timepoint. In the RA group, remission was not associated with any significant difference in mean eGFR; however, mean serum creatinine levels were significantly higher in patients with than without remission at 1 month (*p* = 0.049) and 6 months (*p* = 0.008) after treatment initiation but not at 12 months (*p* = 0.134).

After 12 months of treatment, the proportion of patients with disease relapse was 45.5% in the PsA group and 54.5% in the RA group. Among patients with PsA, those with, as opposed to without, disease relapse at that timepoint had significantly higher levels of mean eGFR (*p* = 0.048) and serum creatinine (*p* = 0.031) at treatment initiation. In patients with RA, there were no significant differences in mean eGFR between those who relapsed and those who did not, but mean serum creatinine levels were significantly higher in patients with than without relapse at 1 month (*p* = 0.009), 3 months (*p* = 0.014), and 6 months (*p* = 0.014) after treatment initiation.

Next, we determined whether the differences in mean serum creatinine levels between patients with/without remission at different timepoints could be confounded by treatment type. Our analysis in PsA patients showed no association between creatinine levels and ACE inhibitors (*n* = 5) (1 month: *p* = 0.125, 3 months: *p* = 0.333, 6 months: *p* = 0.312, 12 months: *p* = 0.125, last assessment: *p* = 0.900), methotrexate or TNF inhibitors (*n* = 18) (1 month: *p* = 0.112, 3 months: *p* = 0.780, 6 months: *p* = 0.231, 12 months: *p* = 0.501, last assessment: *p* = 0.523), or IL-17 inhibitors (*n* = 5) (1 month: *p* = 0.690, 3 months: *p* = 0.801, 6 months: *p* = 0.607, 12 months: *p* = 0.490, last assessment: *p* = 0.611). Only one patient was treated with steroids, and another patient was under a CsA regimen. Pooling all treatments into a covariate did not change these findings. Similarly, in RA patients, during the follow-up, there was no association between creatinine levels and treatment with steroids (*n* = 13) (1 month: *p* = 0.090, 3 months: *p* = 0.118, 6 months: *p* = 0.325, 12 months: *p* = 0.658, last assessment: *p* = 0.089), methotrexate or TNF inhibitors (*n* = 17) (1 month: *p* = 0.324, 3 months: *p* = 0.333, 6 months: *p* = 0.433, 12 months: *p* = 0.568, last assessment: *p* = 0.599), or other biologics (abatacept *n* = 4, rituximab *n* = 2, or IL-6 inhibitors, *n* = 2) (1 month: *p* = 0.111, 3 months: p = 0.123, 6 months: *p* = 0.550, 12 months: *p* = 0.430, last assessment: *p* = 0.390). A similar picture was observed for ACE inhibitors (*n* = 3) (all *p* > 0.050). No patients were on immunosuppressive treatment (mycophenolate mofetil, azathioprine, cyclosporine A). Pooling all treatments and including them as a covariate did not change these findings. Equivalent findings were retrieved when the eGFR was entered as the dependent variable in the analyses (all *p* > 0.050), hence reinforcing our findings.

## 4. Discussion

This retrospective study of patients with PsA and RA in a real-world clinical practice demonstrated few differences between disease groups with respect to age, medical history, and renal function parameters during 12 months of treatment. The prevalence of abnormalities in renal parameters did not significantly differ between disease groups, ranging from 38.5% to 58.3% in patients with PsA and from 45.5% to 54.5% in those with RA. In the PsA group, the distribution of mean eGFR and serum creatinine was not significantly different between patients with versus without clinical remission at corresponding timepoints but was significantly higher at treatment initiation in those with versus without relapse. In the RA group, mean serum creatinine levels were significantly higher in patients with versus without clinical remission at 1 and 6 months after treatment initiation and in patients with versus without relapse after 1, 3, and 6 months of treatment, whereas mean eGFR showed no significant difference in RA patients with versus without clinical remission or relapse. Our analyses showed no association between treatment and creatinine levels. Thus, our results seem to suggest that the association between kidney parameter abnormalities and disease activity is related to the degree of control of the disease, regardless of the therapeutic agent used to achieve the remission/relapse state. In addition, we ruled out the confounding effects of gender, BMI, and arterial hypertension.

The findings of our study are consistent with those of a previous analysis by Haroon and colleagues, which compared renal function parameters in patients with RA and those with seronegative arthritis (91% of whom had PsA) [16]. Haroon and colleagues found no difference in mean eGFR between patients with RA and those with seronegative arthritis (83 vs. 97 mL/min/1.73 m^2^) and no between-group difference in the proportion of patients with reduced eGFR (19% vs. 16%). No individual CVD diagnosis was significantly more prevalent in the RA group than the seronegative arthritis group; however, the prevalence of hypertension was numerically higher in patients with RA than in those with seronegative arthritis (19% vs. 7%), and when all CVD diagnoses were considered together (i.e., ischemic heart disease, transient ischemic attack, congestive heart failure, peripheral vascular disease, and hypertension), the incidence of any CVD was significantly higher in the RA group than in the seronegative arthritis group (32% vs. 12%; *p* = 0.028) [16]. In addition, that study found that CKD was significantly associated with the duration of arthritis and high CRP levels [16], whereas cross-sectional studies have shown that arthritis risk factors are not significant predictors of CKD in patients with RA [14,15].

In our study, patients with PsA had a significantly higher mean BMI and a greater incidence of hypertension than patients with RA. In this respect, our data are consistent with those from a Norwegian nationwide assessment of cardiovascular risk factors in patients with inflammatory joint diseases, which also showed a higher BMI and greater incidence of hypertension compared with other inflammatory joint diseases, including RA and axial spondyloarthropathies [22].

Serum creatinine levels at the time of diagnosis with renal dysfunction are a marker for declining renal function in patients with RA [23], suggesting that physicians should monitor renal function more closely in those with elevated serum creatinine levels.

The limitations of this study include the small number of participants, which limited the power to detect significant differences in patient characteristics between disease groups or treatment received [24,25]. However, it must be noted that the PsA and RA groups under analysis are representative of real-world populations. There was also a lack of biopsy data to enable identification of specific types and causes of renal involvement. Further, the retrospective nature of the study design means that there was a potential for selection bias, and the study was reliant on accurate recording of patient data and on what was recorded in electronic medical records. For example, PsA disease activity was assessed by the DAS28 score only, and no data on skin involvement, enthesitis, and dactylitis were available in the electronic patient records. In addition, since remission is the target of T2T strategies, and the study was conducted in the real-world setting, we did not assess the relationship between renal function parameters and disease severity/degree of inflammatory activity. Our aim was to analyze the effect of remission itself on renal function and not the association between renal function and the range of parameters related to disease activity. Larger prospective studies are needed to validate these results and evaluate the potential role of other confounders.

## 5. Conclusions

In our single-center retrospective study, the prevalence of renal parameter abnormalities during 12 months of treatment was similar in patients with PsA and RA. There were also minimal differences in clinical characteristics between patients with PsA and RA, although patients with PsA had a slightly higher mean BMI and a greater incidence of hypertension than those with RA. In patients with RA, clinical remission and relapse did not correlate with the mean eGFR at the corresponding timepoint, whereas in patients with PsA, disease relapse was associated with renal function parameter abnormalities at treatment initiation.

## Figures and Tables

**Table 1:** Clinical and demographic characteristics of patients at baseline.

	All Patients (*n* = 45)	PsA Patients (*n* = 23)	RA Patients (*n* = 22)	*p*-Value ^a^
Mean (SD) age, years	55.6 (15.9)	57.4 (10.2)	54.3 (19.3)	0.664 ^b^
Sex, *n* (%)				
Male	10 (22.2)	6 (26.1)	4 (18.2)	0.722
Female	35 (77.8)	17 (73.9)	18 (81.8)	
Medical history, ^c^ *n* (%)				
Hypertension	11 (25.0)	7 (31.8)	4 (18.2)	0.488
Diabetes mellitus	5 (11.4)	3 (13.6)	2 (9.1)	1.00
Autoimmune disease	4 (9.1)	3 (13.6)	1 (2.3)	0.607
Kidney failure	1 (2.3)	1 (2.3)	0	1.00
Smoking, ^c^ *n* (%)				
Never	35 (79.5)	21 (95.5)	14 (63.6)	0.029 ^d^
Former	3 (6.8)	0	3 (13.6)	
Current	6 (13.6)	1 (4.5)	5 (22.7)	
Anti-CCP autoantibody positive, *n* (%)		0	18 (80.7)	–
RF positive, *n* (%)		0	16 (75.0)	–
Mean (SD) ESR, mm/h		10.0 (8.7)	35.0 (17.0)	–
Mean (SD) CRP, mg/dL		9.0 (5.7)	40.0 (20.7)	–
Concurrent hypertension diagnosis, ^e^ *n* (%)	11 (25.6)	9 (42.9)	2 (9.1)	0.016
Concomitant ACE inhibitors or ARBs, ^e^ *n* (%)	8 (18.6)	5 (23.8)	3 (13.6)	0.457

^a^ Fisher’s exact test, two-sided significance level, unless otherwise indicated. ^b^ Mann–Whitney U test. ^c^ Data missing for one patient with PsA; therefore, overall *n* = 44 and PsA group *n* = 22 were used to calculate percentages. Referred to previous or current diagnosis. ^d^ Pearson chi-squared test. ^e^ Data missing for two patients with PsA; therefore, overall *n* = 43 and PsA group *n* = 21 were used to calculate percentages. Referred to current (existing) diagnosis. ACE, angiotensin-converting enzyme; ARB, angiotensin receptor blocker; PsA, psoriatic arthritis; RA, rheumatoid arthritis; SD, standard deviation.

**Table 2:** Distribution of physical characteristics of the two disease groups at baseline.

Variable, Mean (±SD)	PsA Patients (*n* = 23)	RA Patients (*n* = 22)	*p*-Value ^a^
Weight, kg	81.9 (11.2)	67.6 (14.3)	0.001
Height, m	1.7 (0.1)	1.6 (0.1)	0.015
BMI, kg/m^2^	28.8 (3.5)	25.0 (6.7)	0.010

^a^ Linear regression model adjusted for sex. BMI, body mass index; PsA, psoriatic arthritis; RA, rheumatoid arthritis; SD, standard deviation.

**Table 3:** Renal function measurements in the two disease groups.

Variable, Mean ± SD	PsA Patients (*n* = 23)	RA Patients (*n* = 22)	*p*-Value ^a^
Serum creatinine level, mg/dL			
At treatment initiation	0.83 (0.20)	0.79 (0.19)	0.528
At 1 month	0.78 (0.17)	0.83 (0.18)	0.302
At 3 months	0.83 (0.17)	0.83 (0.16)	0.614
At 6 months	0.89 (0.30)	0.78 (0.15)	0.466
At 12 months	0.88 (0.29)	0.82 (0.19)	0.749
At the last assessment	0.88 (0.20)	0.83 (0.17)	0.585
eGFR, mL/min/1.73 m^2^			
At treatment initiation	87.93 (19.62) ^b^	102.87 (45.88)	0.510
At 1 month	92.39 (18.57) ^c^	93.86 (32.13)	0.699
At 3 months	86.19 (16.88) ^d^	92.89 (29.37)	0.939
At 6 months	84.85 (23.18) ^e^	100.38 (35.78)	0.345
At 12 months	86.65 (26.88) ^f^	99.42 (47.99)	0.807
At the last assessment	82.29 (16.05) ^g^	96.02 (42.12)	0.624

^a^ Mann–Whitney U test. eGFR, estimated glomerular filtration rate; PsA, psoriatic arthritis; RA, rheumatoid arthritis; SD, standard deviation. ^b^ *n* = 18; ^c^ *n* = 13; ^d^ *n* = 15, ^e^ *n* = 12, ^f^ *n* = 11, ^g^ *n* = 16.

## Data Availability

Data sharing not applicable.

## Supplementary Materials

The following supporting information can be downloaded at: https://www.mdpi.com/article/10.3390/jcm11041029/s1, Figure S1: Distribution of estimated glomerular filtration rate in patients with psoriatic arthritis (PsA) or rheumatoid arthritis (RA) over 12 months of treatment stratified according to chronic kidney disease stage; Table S1: Regression analysis of creatinine levels using gender, BMI and hypertension as covariates at all timepoints in the psoriatic arthritis (PsA) group; Table S2: Regression analysis of creatinine levels using gender, BMI and hypertension as covariates at all timepoints in the rheumatoid arthritis (RA) group.

## References

[1] Kapoor T., Bathon J. (2018). Renal manifestations of rheumatoid arthritis. Rheum. Dis. Clin. N. Am..

[2] Ponticelli C., Doria A., Moroni G. (2020). Renal disorders in rheumatologic diseases: The spectrum is changing (part 2. Arthridides). J. Nephrol..

[3] Fayed A., Shaker A., Hamza W.M., Wadie M. (2019). Spectrum of glomerulonephritis in Egyptian patients with rheumatoid arthritis: A University Hospital experience. Saudi J. Kidney Dis. Transplant..

[4] Ayar Y., Ersoy A., Oksuz M.F., Ocakoglu G., Vuruskan B.A., Yildiz A., Isiktas E., Oruc A., Celikci S., Arslan I. (2017). Clinical outcomes and survival in AA amyloidosis patients. Rev. Bras. Reumatol. Engl. Ed..

[5] Engineer D.P., Kute V.B., Patel H.V., Shah P.R. (2018). Clinical and laboratory profile of renal amyloidosis: A single-center experience. Saudi J. Kidney Dis. Transplant..

[6] Laiho K., Tiitinen S., Kaarela K., Helin H., Isomäki H. (1999). Secondary amyloidosis has decreased in patients with inflammatory joint disease in Finland. Clin. Rheumatol..

[7] Pamuk N., Kalyoncu U., Aksu K., Omma A., Pehlivan Y., Çağatay Y., Küçükşahin O., Dönmez S., Çetin G.Y., Mercan R. (2016). A multicenter report of biologic agents for the treatment of secondary amyloidosis in Turkish rheumatoid arthritis and ankylosing spondylitis patients. Rheumatol. Int..

[8] Pamuk O.N., Dönmez S., Pamuk G.E., Puyan F.O., Keystone E.C. (2013). Turkish experience in rheumatoid arthritis patients with clinical apparent amyloid deposition. Amyloid.

[9] Oweis A.O., Alawneh K.M., Alshelleh S.A., Alnaimat F., Alawneh D., Zahran D.J. (2020). Renal dysfunction among rheumatoid arthritis patients: A retrospective cohort study. Ann. Med. Surg..

[10] Sarnak M.J., Levey A.S., Schoolwerth A.C., Coresh J., Culleton B., Hamm L.L., McCullough P.A., Kasiske B.L., Kelepouris E., Klag M.J. (2003). Kidney disease as a risk factor for development of cardiovascular disease: A statement from the American Heart Association Councils on kidney in cardiovascular disease, high blood pressure research, clinical cardiology, and epidemiology and prevention. Hypertension.

[11] Chiu H.-Y., Huang H.-L., Li C.-H., Chen H.-A., Yeh C.-L., Chiu S.-H., Lin W.-C., Cheng Y.-P., Tsai T.-F., Ho S.-Y. (2015). Increased risk of chronic kidney disease in rheumatoid arthritis associated with cardiovascular complications—A national population-based cohort study. PLoS ONE.

[12] Jankowski J., Floege J., Fliser D., Böhm M., Marx N. (2021). Cardiovascular disease in chronic kidney disease: Pathophysiological insights and therapeutic options. Circulation.

[13] Bressolle F., Bologna C., Kinowski J.-M., Sany J., Combe B. (1998). Effects of moderate renal insufficiency on pharmacokinetics of methotrexate in rheumatoid arthritis patients. Ann. Rheum. Dis..

[14] Daoussis D., Panoulas V.F., Antonopoulos I., John H., Toms T., Wong P., Nightingale P., Douglas K.M.J., Kitas G.D. (2010). Cardiovascular risk factors and not disease activity, severity or therapy associate with renal dysfunction in patients with rheumatoid arthritis: Table 1. Ann. Rheum. Dis..

[15] Mori S., Yoshitama T., Hirakata N., Ueki Y. (2017). Prevalence of and factors associated with renal dysfunction in rheumatoid arthritis patients: A cross-sectional study in community hospitals. Clin. Rheumatol..

[16] Haroon M., Adeeb F., Devlin J., Ogradaigh D., Walker F. (2011). A comparative study of renal dysfunction in patients with inflammatory arthropathies: Strong association with cardiovascular diseases and not with anti-rheumatic therapies, inflammatory markers or duration of arthritis. Int. J. Rheum. Dis..

[17] Taylor W., Gladman D., Helliwell P., Marchesoni A., Mease P., Mielants H., CASPAR Study Group (2006). Classification criteria for psoriatic arthritis: Development of new criteria from a large international study. Arthritis Rheum..

[18] Aletaha D., Neogi T., Silman A.J., Funovits J., Felson D.T., Bingham C.O., Birnbaum N.S., Burmester G.R., Bykerk V.P., Cohen M.D. (2010). 2010 Rheumatoid arthritis classification criteria: An American College of Rheumatology/European League Against Rheumatism collaborative initiative. Arthritis Rheum..

[19] van der Heijde D.M., van’t Hof M., van Riel P.L., van de Putte L.B. (1993). Development of a disease activity score based on judgment in clinical practice by rheumatologists. J. Rheumatol..

[20] Cockcroft D.W., Gault H. (1976). Prediction of creatinine clearance from serum creatinine. Nephron.

[21] Atzeni F., Gerratana E., Masala I.F., Bongiovanni S., Sarzi-Puttini P., Rodríguez-Carrio J. (2021). Psoriatic arthritis and metabolic syndrome: Is there a role for disease modifying anti-rheumatic drugs?. Front. Med..

[22] Wibetoe G., Ikdahl E., Rollefstad S., Olsen I.C., Bergsmark K., Kvien T.K., Salberg A., Soldal D.M., Bakland G., Lexberg Å. (2017). Cardiovascular disease risk profiles in inflammatory joint disease entities. Arthritis Res. Ther..

[23] Zhang T., Liang S., Feng X., Li M., Zhou H., Zeng C., Zhang J., Cheng Z. (2020). Spectrum and prognosis of renal histopathological lesions in 56 Chinese patients with rheumatoid arthritis with renal involvement. Clin. Exp. Med..

[24] Mikhaylov D., Hashim P.W., Nektalova T., Goldenberg G. (2019). Systemic psoriasis therapies and comorbid disease in patients with psoriasis: A review of potential risks and benefits. J. Clin. Aesthetic Dermatol..

[25] Stokes M.B., Foster K., Markowitz G.S., Ebrahimi F., Hines W., Kaufman D., Moore B., Wolde D., D’Agati V.D. (2005). Development of glomerulonephritis during anti-TNF-α therapy for rheumatoid arthritis. Nephrol. Dial. Transplant..

