# Supplementary material for: Frequency of Renal Function Parameter Abnormalities in Patients with Psoriatic Arthritis and Rheumatoid Arthritis: Real-World Evidence from Clinical Practice
Source: J Clin Med. 2022 Feb 16;11(4):1029. doi: 10.3390/jcm11041029 (PMC8880606; doi:10.3390/jcm11041029)
Supplement: Supplementary file 1 [file jcm-11-01029-s001.zip › jcm-1549683_Supplementary Tables.pdf]

Supplementary Table S1. Regression analysis of creatinine levels using gender, BMI and hypertension as covariates at all timepoints in the PsA group.

Timepoint	Gender		BMI		HTA	
	Beta [95% CI]	p-value	Beta [95% CI]	p-value	Beta [95% CI]	p-value
Baseline	-33.857 [-57.144, -10.570]	0.018	3.361 [-0.199, 6.524]	0.089	-10.530 [-31.027, 9.960]	0.289
1 month	-20.195 [-45.397, 5.007]	0.104	5.162 [-0.600, 9.724]	0.080	-9.913 [-36.639, 16.813]	0.428
3 months	-15.391 [-40.503, 9.722]	0.207	3.539 [-0.300, 7.378]	0.068	-7.506 [-30.030, 15.015]	0.482
6 months	-24.740 [-51.139, 1.660]	0.063	7.716 [-2.909, 12.523]	0.500	-8.683 [-46.751, 29.383]	0.618
12 months	-31.560 [-71.358, 8.238]	0.105	6.312 [-0.973, 13.598]	0.081	10.001 [-36.589, 56.593]	0.634
Last assessment	-20.733 [-41.846, 0.380]	0.054	-0.704 [-5.017, 3.609]	0.736	-4.063 [-22.916, 14.783]	0.651

BMI, body mass index; CI, confidence interval; HTA, arterial hypertension

Supplementary Table S2. Regression analysis of creatinine levels using gender, BMI and hypertension as covariates at all timepoints in the RA group.

Timepoint	Gender		BMI		HTA	
	**Beta [95% CI]**	**p-value**	**Beta [95% CI]**	**p-value**	**Beta [95% CI]**	**p-value**
Baseline	-8.919 [-45.277, 63.114]	0.734	-2.259 [-6.848, 2.331]	0.316	-15.505 [-87.871, 56.861]	0.660
1 month	-7.523 [-46.057, 31.011]	0.687	-0.961 [-4.224, 2.303]	0.545	-14.994 [-65.448, 35.458]	0.542
3 months	-0.801 [-34.716, 36.319]	0.973	-0.703 [-3.711, 2.305]	0.630	-24.829 [-69.927, 20.276]	0.264
6 months	19.374 [-22.777, 61.525]	0.348	-1.052 [-4.621, 2.518]	0.545	-17.973 [-74.071, 38.126]	0.512
12 months	-14.685 [-42.591, 71.960]	0.598	-1.614 [-6.465, 3.236]	0.494	-21.111 [-96.549, 54.321]	0.566
Last assessment	-8.275 [-59.204, 42.654]	0.738	2.828 [-0.138, 5.697]	0.113	-24.822 [-90.574, 40.925]	0.440

BMI, body mass index; CI, confidence interval; HTA, arterial hypertension
