# Supplementary figures and images for: Frequency of Renal Function Parameter Abnormalities in Patients with Psoriatic Arthritis and Rheumatoid Arthritis: Real-World Evidence from Clinical Practice
Source: J Clin Med. 2022 Feb 16;11(4):1029. doi: 10.3390/jcm11041029 (PMC8880606; doi:10.3390/jcm11041029)

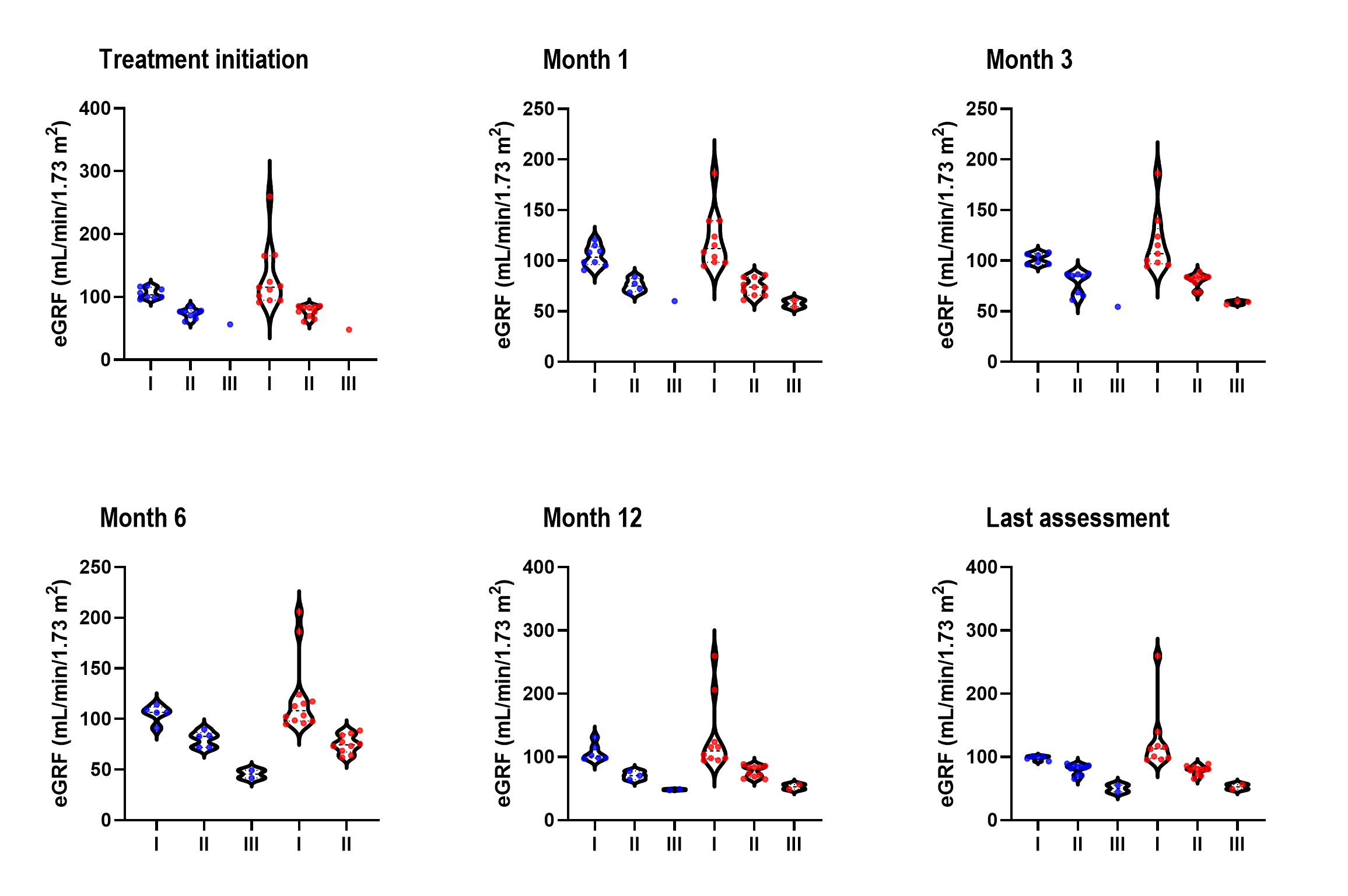

Supplement: Supplementary file 1 [file jcm-11-01029-s001.zip › jcm-1549683_Supplementary Figure S1.png]
